# An integrative approach to assessing effects of a short-term Western diet on gene expression in rat liver

**DOI:** 10.3389/fendo.2022.1032293

**Published:** 2022-10-26

**Authors:** Jaclyn E. Welles, Holly Lacko, Yuka Imamura Kawasawa, Michael D. Dennis, Leonard S. Jefferson, Scot R. Kimball

**Affiliations:** ^1^ Department of Cellular and Molecular Physiology, Penn State College of Medicine, Hershey, PA, United States; ^2^ Department of Pharmacology, Penn State College of Medicine, Hershey, PA, United States

**Keywords:** diet-induced obesity, nonalcoholic fatty liver disease (NAFLD), mRNA translation, mTORC1, gene regulation, liver, metabolism

## Abstract

Consumption of a diet rich in saturated fatty acids and carbohydrates contributes to the accumulation of fat in the liver and development of non-alcoholic steatohepatitis (NASH). Herein we investigated the hypothesis that short-term consumption of a high fat/sucrose Western diet (WD) alters the genomic and translatomic profile of the liver in association with changes in signaling through the protein kinase mTORC1, and that such alterations contribute to development of NAFLD. The results identify a plethora of mRNAs that exhibit altered expression and/or translation in the liver of rats consuming a WD compared to a CD. In particular, consumption of a WD altered the abundance and ribosome association of mRNAs involved in lipid and fatty acid metabolism, as well as those involved in glucose metabolism and insulin signaling. Hepatic mTORC1 signaling was enhanced when rats were fasted overnight and then refed in the morning; however, this effect was blunted in rats fed a WD as compared to a CD. Despite similar plasma insulin concentrations, fatty acid content was elevated in the liver of rats fed a WD as compared to a CD. We found that feeding had a significant positive effect on ribosome occupancy of 49 mRNAs associated with hepatic steatosis (e.g., LIPE, LPL), but this effect was blunted in the liver of rats fed a WD. In many cases, changes in ribosome association were independent of alterations in mRNA abundance, suggesting a critical role for diet-induced changes in mRNA translation in the expression of proteins encoded by those mRNAs. Overall, the findings demonstrate that short-term consumption of a WD impacts hepatic gene expression by altering the abundance of many mRNAs, but also causes wide-spread variation in mRNA translation that potentially contribute to development of hepatic steatosis.

## Introduction

Nonalcoholic fatty liver disease (NAFLD) refers to a spectrum of conditions ranging from nonalcoholic liver (NAFL) to nonalcoholic steatohepatitis (NASH) and is the most common cause of chronic liver failure worldwide and is the second leading indicator for liver transplant (1, 2). It is defined as the presence of steatosis in >5% of hepatocytes in the absence of excessive alcohol consumption (3). The development of NAFLD is highly and positively associated with obesity and type 2 diabetes. Indeed, overnutrition is one of the leading causes of NAFLD. Moreover, a subset of patients (3-15%) develop NASH, resulting in fibrosis, liver injury, and in some cases (4-27%) the development of hepatocellular carcinoma (HCC). The accumulation of other lipids including triglycerides, diacylglycerols, and ceramides likely contributes to the progression from NAFL to more aggressive forms such as NASH (4). In addition, the accumulation of fat in the liver is exacerbated by the development of insulin resistance in adipose tissue, leading to increased delivery of free fatty acids and glycerol to the liver, placing an additional burden for hepatic health (5, 6). There are a number of rodent models of NAFLD, many of them focused on dietary manipulation as a mechanism for inducing fat accumulation in the liver (7, 8). For example, the excess supply of free fatty acids provided by diets with elevated fat content, i.e., “high-fat” diets (HFD), leads not only to triglyceride accumulation in the liver but also to the development of insulin resistance and obesity. Variations of such diets are designed to more closely mimic the diet of individuals in Western society, e.g., by inclusion of cholesterol and/or sucrose (i.e., a “Western” diet).

Previous studies have shown that the activity of the protein kinase mechanistic target of rapamycin (mTOR) is altered in the liver of mice fed either a high-fat or Western diet compared to a control diet (9–13). Activation of mTOR in complex 1 (mTORC1) in the liver upon refeeding leads to a reduction in ketogenesis, followed by activation of lipid and fatty acid metabolism pathways, such as the *de novo* lipogenesis pathway (14, 15). Primarily occurring in the liver and adipose tissue following a meal, *de novo* lipogenesis is the process by which circulating carbohydrates are converted into fatty acids and triglycerides (14). *De novo* lipogenesis is also elevated in patients suffering from NAFLD (16). Interestingly, activation of hepatic mTORC1 leads to upregulated expression of lipogenic genes (17–19), promoting *de novo* lipogenesis and hepatic steatosis in the liver, a metabolic phenotype also observed in patients with NAFL and non-alcoholic steatohepatitis (NASH) (20). mTORC1 also modulates lipid metabolism through regulation of sterol and regulatory element binding protein-1c (SREBP-1c), a basic helix-loop-helix transcription factor that controls the expression of genes required for cholesterol and fatty acid metabolism, as well as phospholipid synthesis (21–24). Interestingly, rapamycin-induced inhibition of mTORC1 activity leads to the acute abrogation of downstream SREBP-1c effectors, e.g., acetyl-CoA carboxylase, in primary rat hepatocytes (25). Notably, in animal models of obesity, rapamycin administration attenuates inflammation and inhibited progression of atherosclerotic plaques (26). Furthermore, mice lacking the mTORC1 inhibitor tuberous sclerosis complex 1 in the liver, which leads to insulin-independent activation of mTORC1, are resistant to diet-induced obesity, hepatic steatosis, and hypercholesteremia (14, 27).

Both preclinical and clinical studies have provided evidence that mTORC1 is activated in NAFL and NASH-induced hepatocellular carcinoma (HCC), though its exact role in promoting HCC is unknown (28). Previous studies from our laboratory demonstrated that consumption of a 60% HFD for 2-weeks leads to attenuation of the activation state of mTORC1 (29). In contrast, chronic consumption of a HFD (e.g., 8-weeks or 16-weeks) significantly enhances mTORC1 expression and activation state in rats, correlating directly with increases in hepatic steatosis when compared to control-diet fed rats (30). Enhanced mTORC1 activation is also observed in livers of rats fed a diet with 32.5% kcal from fat and 20% kcal from sucrose for 4-weeks when compared to CD-fed rats (31). Moreover, in recent studies wherein rats were provided drinking water supplemented with 10% (w/v) fructose, mTOR autophosphorylation on Ser2481 is upregulated (12, 32–34). In contrast, mTORC1 activity is acutely, i.e., within 30 min, repressed in the liver in response to oral administration of fructose (12). Together, these findings highlight that both the length of time on a diet, as well as its composition significantly influences the regulation of mTORC1 activity in the liver.

Interestingly, mTORC1 signaling is closely associated with the regulation of cap-dependent mRNA translation in the liver (35–39). Studies have demonstrated that following a period of food deprivation, feeding-induced activation of mTORC1 enhances the association of ribosomes with mRNAs that encode proteins relating to the translation machinery (e.g., ribosomal proteins, and translation elongation and initiation factors) (40, 41). A number of studies have assessed the effects of consumption of a WD on changes in the transcriptome, however, only a few studies have investigated the effects of consumption of a HFD or WD on the translation of mRNAs encoding proteins associated with metabolic disorders (42, 43), and in those studies animals were fed a HFD for 8 or more weeks.

The hypothesis tested in the present study was that short-term, i.e., two-week, consumption of a Western diet alters the genomic and translatomic profile of the liver in association with changes in mTORC1 activity, and that such alterations are, in part, causative in the development of NAFLD. We chose to feed the animals a WD for two weeks because previous studies (e.g. 44) have shown that while feeding a HFD for 2 weeks results in a small, but significant increase in liver triglyceride content, whereas blood glucose, insulin, leptin, and ALT levels are unchanged, suggesting that the animals are in the early stages of developing NAFL, but have not yet become insulin resistant or developed NAFLD. The results show that a large number of mRNAs, many of which encode for proteins crucial in the maintenance of various hepatic metabolic processes (e.g., lipid and glucose metabolism), exhibit altered expression and/or translation in rats consuming a WD compared to a CD, highlighting the importance in continuing to elucidate how consumption of a HFD or WD leads to alterations in mRNA translation in the liver and the metabolic consequence of such changes.

## Methods

### Animals

The animal protocols for the studies described herein were reviewed and approved by the Institutional Animal Care and Use Committee of the Pennsylvania State University College of Medicine (no. PRAMS200946689). Male rats were obtained from Charles River Laboratories (Wilmington, MA), maintained on a 12:12 h light-dark cycle, and provided with water and food *ad libitum*. In the RNA-seq and polysome profiling studies, obesity-prone Sprague Dawley rats (op-SD), initial body weight 160–190 g, were used. For all other studies, Sprague Dawley rats with initial body weights 100–125 g were used. Rats were fed either a control diet (CD; TD 08485), or a Western diet (WD; TD 88137) purchased from Harlan-Teklad (Indianapolis, IN). The composition of the CD was 19.1% kcal protein, 67.9% kcal carbohydrates, and 13% kcal fat (3.7% milk fat; 1.3% soybean oil) (**Supplemental Table 1**). The WD consisted of 15.2% kcal protein, 42.7% kcal carbohydrate, 42% kcal fat (>60% saturated fatty acids (SFAs) from milk-derived fat), 0.2% cholesterol, and 34% sucrose.

In all studies, rats were fed the CD for one week and then randomly divided into two groups with one group continuing to be fed the CD and the other group fed the WD *ad libitum* for two weeks. Thereafter, the rats were fasted overnight for 16-h, and a subset of rats in each group was refed their respective diet for 1-h prior to sacrifice. Rats were anesthetized using isoflurane using an anesthesia induction chamber (EZ Anesthesia). After removal of the liver, rats were euthanized *via* removal of the diaphragm and the heart, as recommended by the American Veterinary Medical Association (45, 46).

### Small animal NMR measurements

A Bruker LF50 Whole Body Composition Analyzer based on Time Domain Nuclear Magnetic Resonance was used to assess whole body fat mass. Prior to scanning, the instrument was calibrated using a lead standard and the standard calibration protocol. Body weights were assessed for each rat prior to scanning.

### Blood analyses

Blood was collected *via* tail vein, and glucose concentrations were measured using a OneTouch Glucose meter following a 6-h fast. Blood was collected from the vena cava of anesthetized rats prior to removal of the liver for serum insulin concentrations using an ultra-sensitive rat insulin ELISA Kit following the manufacturer’s protocol (Crystal Chem, Elk Grove Village, IL; no. 90060).

### Polysome profiling

A sample (~1 g) of the left lobe of the liver was removed, and immediately homogenized in 7 volumes of ice-cold homogenization buffer [50 mM HEPES, pH 7.4, 75 mM KCl, 5 mM MgCl_2_, 250 mM sucrose, 10% Triton X-100, 13% sodium deoxycholate, 100 μg/mL cycloheximide, 2 mM dithiothreitol, and 5 µL/mL RNaseOUT (Invitrogen, no. 1077019)], using a Dounce homogenizer. An aliquot of the homogenate was added to SDS sample buffer, boiled for 5 min, and then frozen and stored at -80°C prior to Western blot analysis as described below. The remainder of the homogenate was transferred to a 50 mL conical tube and centrifuged at 4°C for 10 min at 3,000 x g. Supernatants were then subjected to sucrose density gradient centrifugation at 4°C using a Beckman SW32T rotor at a centrifugal force of 198,200 x g for 3-h and 50 min. Sucrose gradients (20-47% w/v) were made using the “flash/freeze” technique (47–49). After centrifugation, gradients were separated into two fractions using a Density Gradient Fractionation System (ISCO Teledyne) while absorption at 254 nm was continuously recorded. The first fraction corresponded to the portion of the gradient containing mRNAs associated with three or fewer ribosomes (referred to hereafter as the light fraction), and the second fraction corresponded to the portion of the gradient containing mRNAs associated with four or more ribosomes (referred to as the heavy fraction). RNA was extracted from each fraction using 3x volume of TRIzol LS (Invitrogen, California; no. 15596026), followed by an overnight incubation with an equal volume of isopropanol at -20°C, per the manufacturer’s instructions. Heavy fractions were diluted with an equal volume of ribonuclease-free water before RNA extraction, to increase extraction efficiency.

### qRT-PCR

RNA was isolated from the light and heavy fractions of sucrose density gradients as described above or was extracted from rat liver using the TRIzol:chloroform method (i.e., 0.2 mL of chloroform per 1 mL of TRIzol) (50). RNA quality and concentration were determined using a NanoDrop ND-1000 Spectrophotometer (Thermo Fisher Scientific, Waltham, MA). For qRT-PCR analysis, RNA (1 µg) was reverse transcribed into cDNA using a High-Capacity cDNA Reverse Transcription Kit (Applied Biosystems; no. 4374966) and the resulting cDNA was then subjected to qRT-PCR analysis using SYBR Green (Quantitect, Germany; no. 204143) and an ABI QuantStudio 12K Flex system. All primers were purchased from Integrated DNA Technologies and the sequences are listed in **Supplemental Table 2**.

### RNAseq and Riboseq analyses

RNA was isolated as described above, and 20 µg of RNA from the livers of three rats/condition, i.e., rats fed either the CD or WD, or the light and heavy fractions from sucrose density gradients from three rats/condition, were combined prior RNAseq analysis. RNA quality was assessed using an Agilent 2100 Bioanalyzer in the Penn State College of Medicine Genome Sciences Core (RRID : SCR021123). Library preparation from each RNA fraction was performed using a KAPA RNA HyperPrep Kit with RiboErase (Roche Molecular Systems; no. KK8560) according to the manufacturer’s instructions. Briefly, first-strand DNA was synthesized using random primers and the RNA:cDNA hybrid was converted to double-stranded cDNA and dAMP was added to the 3’-ends. Adapters were then ligated to the 3’-dAMP library fragments using the KAPA Single-Indexed Adapter kit (KAPA Biosystems; no. KR1317). The resulting libraries were amplified, and the quality was assessed by electrophoresis followed by RNAseq analysis using an Illumina Novaseq in the Penn State College of Medicine Genome Sciences Core. The Cufflinks v.2.2.1 pipeline (51) was used to align the reads to the rat Rnor_6.0 genome assembly (TopHat) and to assemble and quantify the expression of transcriptomes (Cufflinks, Cuffmerge and Cuffnorm). The DEGseq R package was used to calculate the differential mRNA expression. For ribosome profiling the protocol described by Ingolia et al. (52) was used: Ribo-Seq trimmed reads were aligned to an rRNA reference using Bowtie, the rRNA alignments were discarded, the non-rRNA reads were aligned to the Rnor_6.0 genome assembly using TopHat and the perfect-match alignments were extracted from the TopHat output. The anota R package (53) was used to calculate the differences in actively translated mRNA levels that are independent of underlying differences in cytosolic mRNA levels. mRNAs were considered to be differentially translated if the ratio of mRNA present in the heavy fraction to that present in the light fraction in samples from CD rats compared to the ratio obtained from WD rats was either ≥ 1.5 (i.e., mRNAs with higher ribosome density in CD vs WD rats) or ≤ 0.75 (i.e., mRNAs with lower ribosome density in CD vs WD rats). Pathway analyses were performed using the DAVID (54) and Ingenuity Pathway Analysis (QIAGEN Silicon Valley).

### Western blot analysis

Prior to Western blot analysis, the protein concentration of each sample was assessed using a Pierce Coomassie Blue Plus (Bradford) Assay Kit (ThermoFisher Scientific; no. 23236) and a SpectraMax 190 plate reader. Equal concentrations of protein from each sample were adjusted to the same volume with homogenization buffer and then an equal volume of 2X SDS sample buffer (0.5 M Tris-base, pH 6.8, 25 µl/mL β-mercaptoethanol, and 10% sodium dodecyl sulfate) was added to each sample. After heating at 100°C for 5 min, equal volumes of samples containing equal amounts of protein were loaded into wells of Criterion TGX Precast Gels (BioRad). Following electrophoresis for approximately 60 min at 200V, proteins in the gel were transferred to a polyvinylidene difluoride membrane at 50V for 1.5 h. Membranes were blocked using 5% non-fat dry milk in Tris-buffered saline containing Tween20 (TBS-T) for at least 1 h at room temperature with slow rocking. Membranes were then washed three times with TBS-T for 5 min prior to incubation with primary antibody overnight at 4°C. The next morning, the membrane was washed with TBS-T as described above and then incubated with HRP-labeled goat anti-rabbit antibody for 1 h at room temperature. Information about the antibodies used for Western blot analysis is presented in **Supplemental Table 3**. Membranes were again washed as described above, incubated in Clarity Western ECL Substrate (BioRad, no. 1705060) for 5 min at room temperature, and then imaged using a Fluorchem M imaging system (Protein Simple). ImageJ was used for quantification of the images. To compare signals obtained among blots, the same refed CD sample and refed WD sample were included on each gel and the values for those samples were used to normalize the values for the other samples.

### Statistics and data analysis

Figures were generated and statistical analyses were performed using GraphPad Prism v. 9 (GraphPad Software, Inc., La Jolla, CA). Data are presented as means ± SEM. Student’s t-test was used for analysis when two groups were directly compared. Otherwise, one- or two-way analysis of variance was used with Tukey’s or Dunnett’s correction for multiple comparisons, respectively. Statistical significance was set at p ≤ 0.05.

## Results

**Figure 1:**
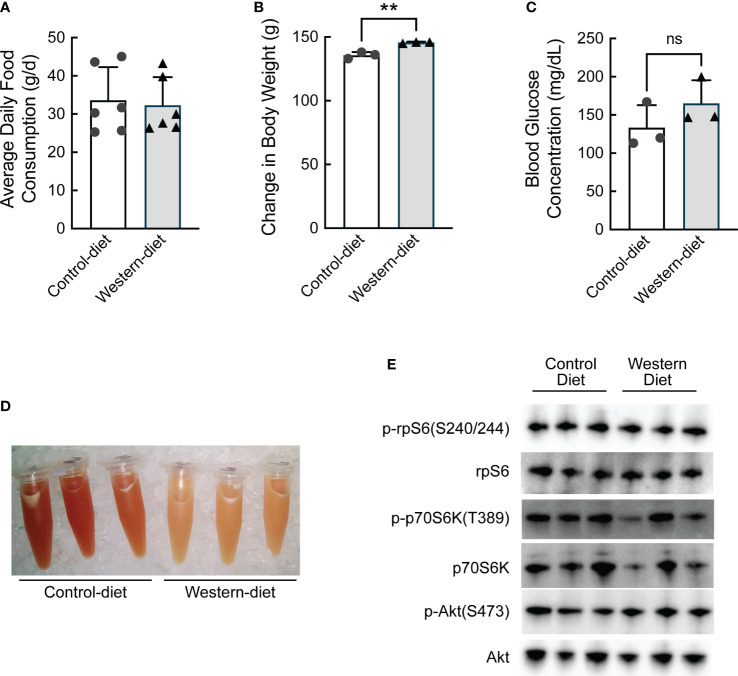
Characterization of the effect of short-term (2 weeks) consumption of a Western diet. **(A)** Average daily food consumption over six 3-d periods, **(B)** change in body weight, **(C)** fasting blood glucose concentration, **(D)** image of liver homogenates, and **(E)** Western blot analysis of liver homogenates. N=3 rats/condition; **p<0.005 vs control diet. ns, not significant.

To assess transcriptional and translation-associated changes in gene expression that manifest during the initial stages of the development of hepatic steatosis, obesity-prone Sprague Dawley (opSD) rats were fed either a CD or a WD for two weeks. Average daily food consumption was the same in both groups of rats (**Figure 1A**). At the end of the two-week period, rats consuming the WD gained slightly, but significantly, more weight compared with rats consuming the CD (**Figure 1B**), likely due to the higher calorie content of the WD compared to the CD (4.5 kcal/g vs. 3.6 kcal/g, respectively). On the penultimate day of the study, rats were fasted for 6 h and blood glucose concentrations were assessed and found to be numerically, but not significantly, higher in rats consuming the WD compared to the CD (**Figure 1C**). The fast was continued overnight and the rats were refed their respective diet for 1 h the next morning. Although hepatic lipid content was not directly assessed, visual inspection of liver homogenates suggested that livers from rats fed the WD contained significantly more lipid compared to controls, with the caveat that some of the lipid in the homogenates was likely present in blood within the liver at the time of homogenization (**Figure 1D**). As assessed by the phosphorylation state of ribosomal protein S6 (rpS6), the 70 kDa ribosomal protein S6 kinase (p70S6K), and Akt, feeding-induced activation of mTORC1 was comparable in the livers of rats fed the two diets (**Figure 1E**).

**Figure 2:**
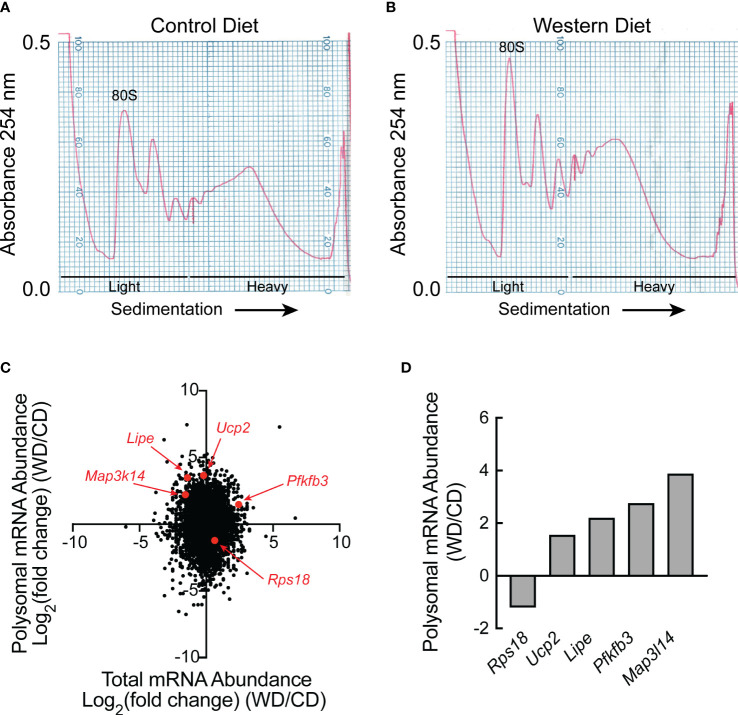
RNAseq and Riboseq analysis of livers of rats fed either a control diet or Western diet. Representative sucrose density gradient profiles from the liver of rats fed either a **(A)** control diet or **(B)** Western diet for 2 weeks. **(C)** Western diet-induced changes in mRNA abundance (x-axis) and the proportion of mRNA in the heavy vs the light polysomal fraction from the sucrose density gradients (y-axis) as assessed by RNAseq and polysome profiling analysis, respectively. For this analysis, equal amounts of RNA from three rats/condition were pooled for RNAseq analysis, and equal amounts of RNA from the sucrose density gradient fractions of three rats/condition were pooled for polysome profiling. **(D)** qRT-PCR analysis of RNA isolated from the heavy and light fractions from the sucrose density gradients.

Liver homogenates were subjected to sucrose density gradient centrifugation, and RNA was extracted from the light and heavy fractions from each gradient as shown in **Figures 2A, B**. Equal amounts of RNA from each of the three light and each of the three heavy fractions were combined and the two samples were subjected to RNAseq analysis, i.e., one combined light and one combined heavy fraction was assessed for each dietary condition. Approximately 11,000 mRNAs were differentially regulated [as demonstrated by log_2_(fold change) (FC)] in the livers of rats fed a WD compared to a CD for two weeks that were either 1) transcriptionally and translationally upregulated, 2) transcriptionally and translationally downregulated, 3) transcriptionally upregulated, but translationally downregulated, or 4) transcriptionally downregulated, but translationally upregulated (**Figure 2C**). Although the number of mRNAs in each quadrant of the graph was similar, it is noteworthy that distribution along the Y-axis (indicating differences in ribosome density, i.e., mRNA translation) was, on average, more pronounced than that along the X-axis (indicating differences in total mRNA abundance). Five mRNAs were selected for validation, and the RNA used for RNAseq analysis was analyzed by qRT-PCR. As shown in **Figure 2D**, the direction of change between dietary conditions was the same in the qRT-PCR analysis as in the RNAseq analysis.

**Figure 3:**
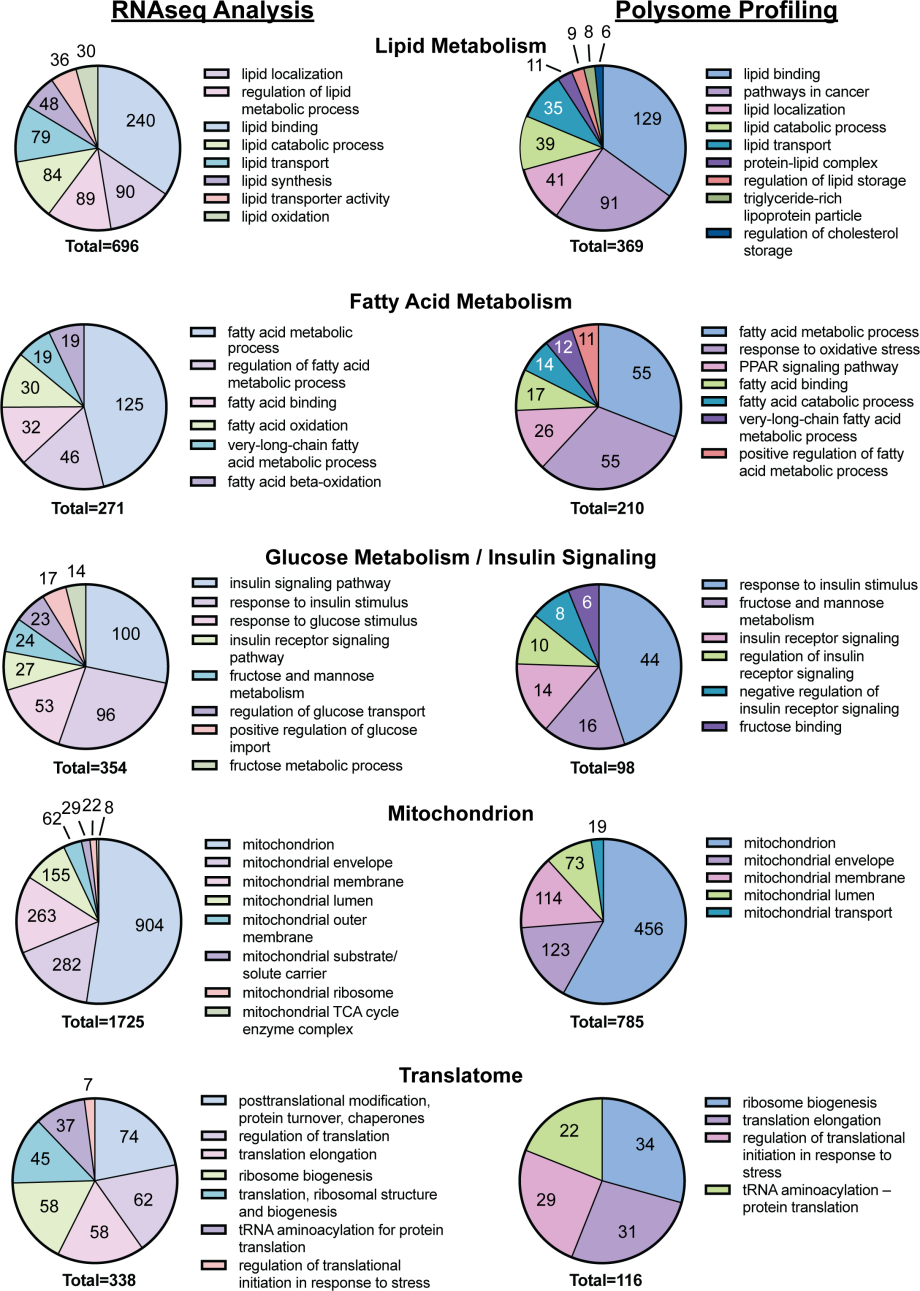
DAVID analysis of the results of the RNAseq and polysome profiling, i.e., Riboseq, analyses from **Figure 2**.

mRNAs that were differentially expressed in the liver of rats fed the CD or the WD as well as mRNAs exhibiting differential ribosome density in the livers of rats fed the two diets were subjected to functional annotation using DAVID. Annotation of mRNA transcripts clustered according to physiological relevance led to the selection of five functional groups associated with a significant number of mRNA transcripts differentially expressed in the livers of WD-fed rats when compared to CD-fed rats (**Figure 3**). Functional clusters identified included lipid metabolism (696 mRNA transcripts including *Lpl* and *Lipe*), fatty acid metabolism (271 mRNA transcripts), glucose metabolism and insulin signaling (354 mRNA transcripts including *Pfkfb3*), the mitochondrion (1,725 mRNA transcripts including *Ucp2*), and the translatome (341 mRNA transcripts including *Rps18*). The mRNAs exhibiting differential ribosome density in the livers of rats fed the two diets also were subjected to functional annotation using DAVID. Annotation analysis of mRNA transcripts differentially associated with ribosomes in response to refeeding a WD compared to CD were categorized into the same five clusters as above. Functional clusters identified include lipid metabolism (369 mRNA transcripts), fatty acid metabolism (210 mRNA transcripts), glucose metabolism and insulin signaling (98 mRNA transcripts), the mitochondrion (785 mRNA transcripts), and the translatome (116 mRNA transcripts).

To further analyze the biological function of genes identified by RNAseq analysis of total and polysome associated mRNAs, functional analysis was performed using Ingenuity Pathway Analysis (**Supplemental Figure 1**). In this analysis, mRNAs exhibiting decreased or increased abundance and decreased or increased ribosome density in the livers of rats fed a WD compared to a CD were assessed. Several processes associated with hepatotoxicity were identified with high significance. Notably, the process with the highest level of significance in all four groups was Liver Hyperplasia/Hyperproliferation. The only other process common to the four groups was Hepatocellular Carcinoma, although Liver Steatosis was identified in three of the four groups.

**Figure 4:**
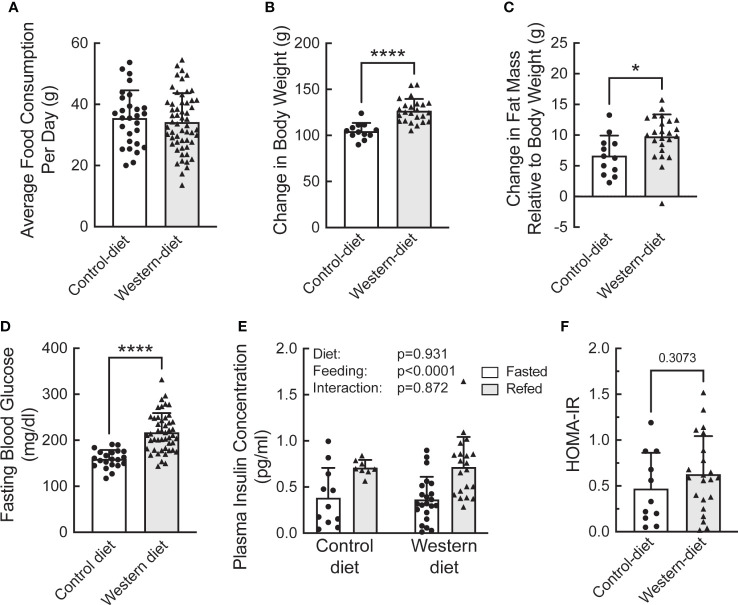
Characterization of the effect of short-term (2 week) consumption of a Western diet on **(A)** average daily food consumption, **(B)** change in body weight, **(C)** change in fat mass, **(D)** fasting blood glucose concentration, **(E)** plasma insulin concentration, and **(F)** HOMA-IR. **(A–D)** N=12 (control diet) or 22 (Western diet) rats/group; **(E)** N=6 (control diet) or 9 (Western diet) rats/group. *p<0.02, **** <0.0001 vs control diet.

To assess the effect of fasting and refeeding on the abundance and ribosome association of mRNAs selected from the study described above, a separate group of Sprague Dawley rats was fed either the CD or WD for two weeks. The rats were then fasted overnight and half the animals in each group were refed for 1 h. The average daily food consumption was the same in the two groups of rats (**Figure 4A**). However, rats fed the WD gained more weight (**Figure 4B**) and had significantly greater fat mass and fasting blood glucose levels compared to rats fed the CD (**Figures 4C, D**, respectively). However, there was no significant difference in either fasting plasma insulin concentrations (**Figure 4E**) or HOMA-IR (Homeostatic Model Assessment for Insulin Resistance; **Figure 4F**) in mice fed a CD compared to mice fed a WD. Moreover, plasma insulin concentrations were higher after refeeding with no difference between rats fed the WD compared to the CD (**Figure 4E**). As expected, the livers of rats fed the WD had significantly higher fatty acid content compared to rats fed the CD, although refeeding had no effect (**Supplemental Figure 2**).

**Figure 5:**
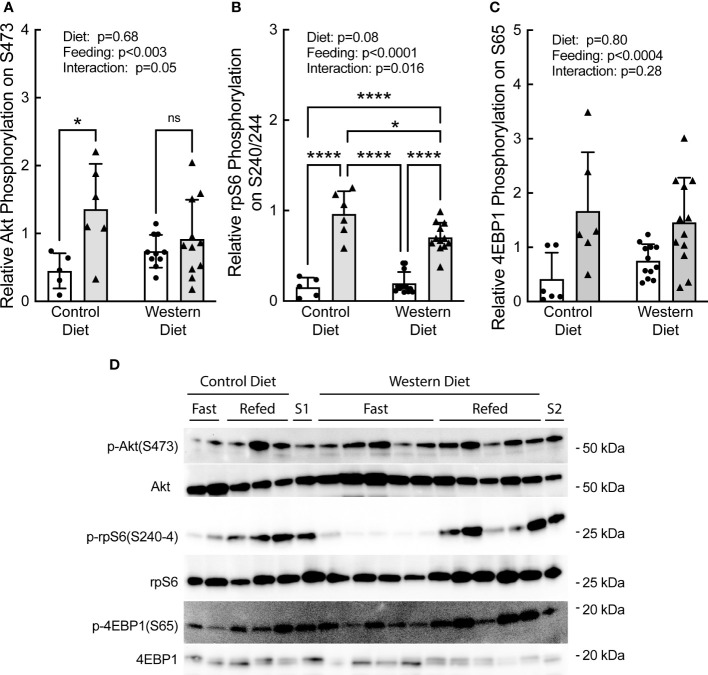
Effect of short-term consumption of a Western diet on hepatic **(A)** Akt, **(B)** ribosomal protein S6 (rpS6), and **(C)** 4E-BP1 phosphorylation. **(D)** Representative blots. S1, liver sample from a refed rat fed a control diet included on each gel as a standard; S2, liver sample from a refed rat fed a WD included on each gel as a standard. N=6-12 rats/group. *p≤0.02, ****p<0.0001. ns, not significant.

Refeeding caused a significant increase in phosphorylation of Akt, rpS6, and 4E-BP1 (**Figures 5A–D**, respectively). Notably, the refeeding-induced increase in Akt and rpS6 phosphorylation was blunted in rats fed the WD compared to rats fed the CD. Together with the data in **Figure 4**, the results suggest that the WD-fed rats may have been in the early stages of developing insulin and/or nutrient resistance.

**Figure 6:**
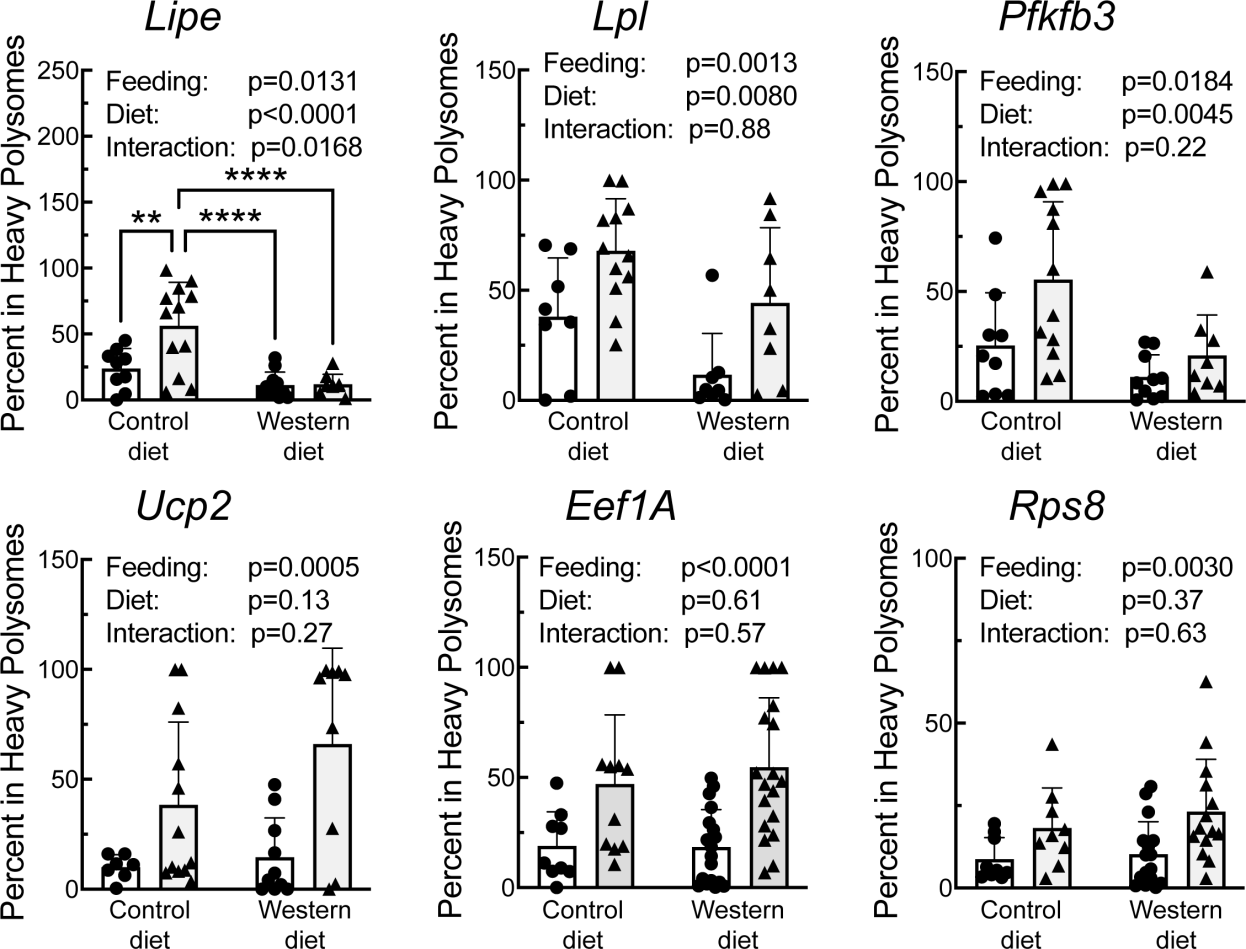
qRT-PCR analysis of the proportion of mRNA in the heavy fraction from sucrose density gradient fraction of livers from rats fed a control diet or a Western diet for 2 weeks. N=8-12 rats/group. **p<0.005, ****p<0.001.

**Figure 7:**
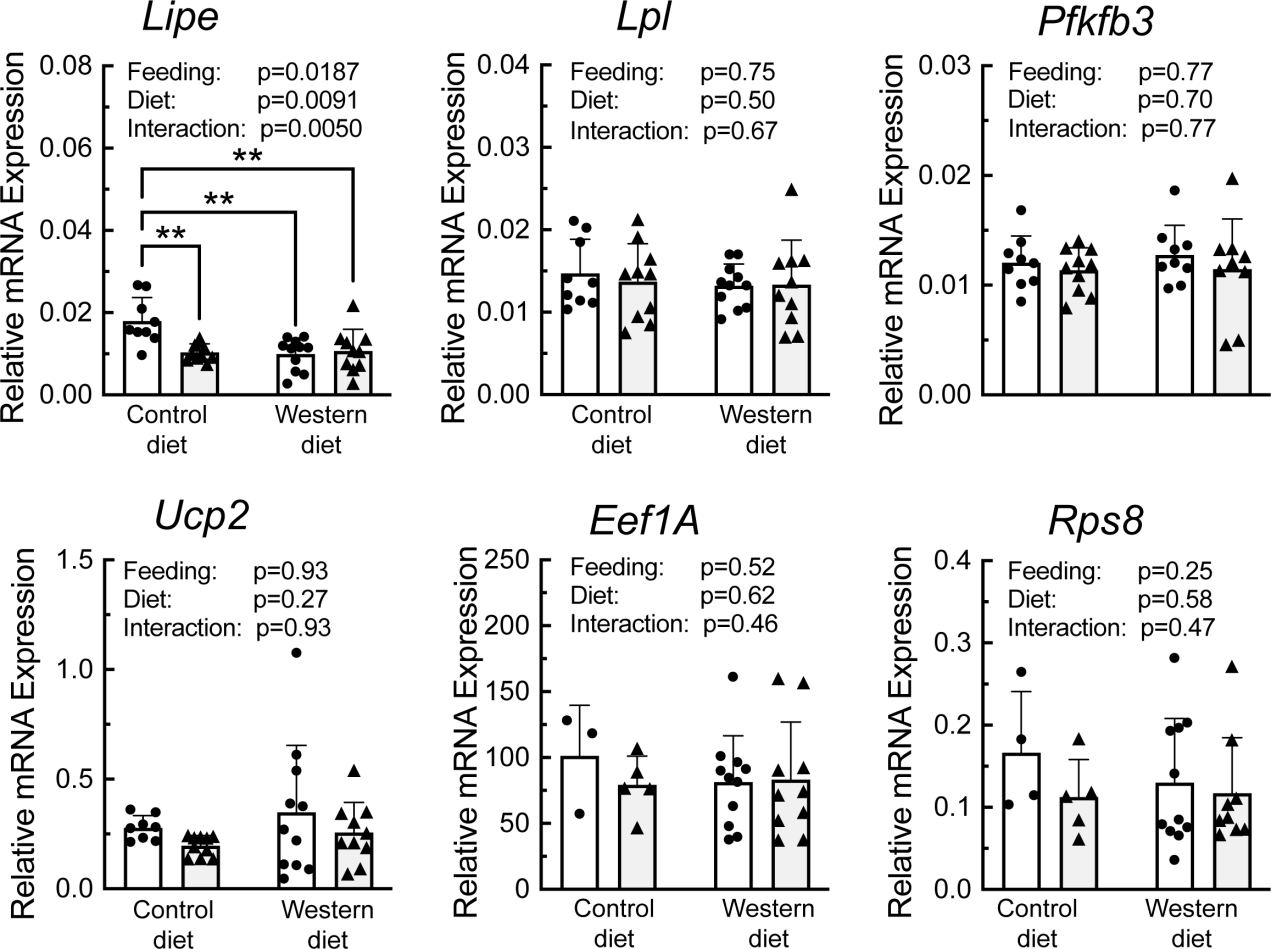
qRT-PCR analysis of mRNA abundance in the liver of rats fed a control diet or Western diet for 2 weeks. n=8-12 rats/group. **p<0.01.

A portion of each liver was subjected to sucrose density gradient centrifugation, and representative profiles for each condition are depicted in **Supplemental Figure 3**. Notably, refeeding was associated with a decrease in the number of 80S ribosomal subunits, and a shift in the profile to the right, i.e., towards mRNAs with more ribosomes attached, consistent with an upregulation of mRNA translation. RNA was isolated from the light and heavy fractions of the sucrose gradients and subjected to RT-PCR analysis for select mRNAs. As shown in **Figure 6**, there was a significant effect of refeeding on the ribosome density on the mRNAs encoding *Lipe*, *Lpl*, *Pfkfb3*, *Ucp2*, *Eef1A*, and *Rps8*. In each case, refeeding promoted a shift of the mRNAs from the light into the heavy fraction, suggesting that refeeding upregulated their translation. Interestingly, there was also a significant effect of diet on ribosome density for the *Lipe*, *Lpl*, and *Pfkfb3* mRNAs, where the refeeding-induced shift of the mRNAs into the heavy fraction was attenuated in the livers of rats fed the WD compared to the CD. There was no significant effect of refeeding or diet on the abundance of the mRNAs encoding *Lpl*, *Pfkfb3*, *Ucp2*, *Eef1A*, or *Rps8* (**Figure 7**). However, *Lipe* mRNA abundance was significantly lower in the livers of rats fed the WD compared to the CD and refeeding was associated with a reduction in *Lipe* mRNA expression in rats fed the control diet.

## Discussion

The number of people who are obese has been steadily increasing for several decades and obesity and its associated complications are now one of the leading causes of death worldwide (55). A major causative factor contributing to the obesity epidemic is consumption of a diet enriched in saturated fatty acids and carbohydrates, i.e., a Western diet (56). One of the consequences of consuming a WD is the accumulation of fat in the liver, which in some cases can progress to NASH, one of the most common reasons for liver transplantation in Western countries (57). To better understand the molecular events leading to development of fatty liver disease, a number of studies have examined the changes in gene expression in the livers of mice and rats fed a WD. Most such studies (9, 58–60) have focused on changes occurring after several weeks or months of WD consumption, and few have assessed changes in gene expression at earlier time points. However, increases in hepatic triglyceride content and lipogenic gene expression manifest in rats fed a HFD for as little as two weeks (61). Moreover, the focus of previous studies has been almost exclusively on changes in the transcriptome without also investigating potential effects of diet on mRNA translation. Yet, changes in both mRNA abundance and translation contribute to establishment of proteostasis in response to a perturbation such as refeeding a fasted animal (62). Consequently, in the present study, the effects of short-term WD consumption on both the transcriptome and translatome in the liver were assessed to delineate some of the early changes in mRNA abundance and translation that occur in response to an obesogenic challenge. The results show that consumption of a WD was associated with changes in mRNA abundance as well as the number of ribosomes bound to numerous mRNAs. Unsurprisingly, functional analysis of WD-induced alterations in the transcriptome and translatome suggested that consumption of a WD had significant effects on both lipid and glucose metabolism as well as insulin signaling in the liver. Moreover, the analysis suggested that consumption of a WD had significant effects on the mitochondria, consistent with the changes in genes involved in lipid and glucose metabolism. The results also showed that ribosome density on 49 mRNAs involved in the development of liver steatosis was increased in the liver of rats fed a WD compared to a CD (**Supplemental Table 4**), indicative of an increase in translation of those mRNAs. In many cases, changes in ribosome association were greater than, and in some cases, independent of alterations in mRNA abundance, suggesting that expression of the proteins encoded by those mRNAs is primarily controlled through translational rather than transcriptional mechanisms.

One of the best studied mechanisms for regulating mRNA translation involves reversible changes in mTORC1 activity. For example, activation of mTORC1 has selective effects on translation of a subset of mRNAs, many of which have a terminal oligopyrimidine (TOP) motif at the 5’-end of the message (63). More recent studies (e.g 36, 64), showed that mTORC1 also promotes the translation of mRNAs lacking a classical TOP motif, e.g., mRNAs with a uridine immediately after the m^7^GTP cap instead of cytosine, or mRNAs with an oligopyrimidine tract within four nucleotides of the transcription start site, i.e., TOP-like mRNAs (36). In the present study, three of the mRNAs that exhibited enhanced ribosome density in response to refeeding, i.e., *Eef1a*, *Rps8* and *Lpl*, possess classical TOP motifs and another mRNA, *Lipe*, possesses a TOP-like motif based on the sequence from the NCBI website (**Supplemental Table 5**; https://www.ncbi.nlm.nih.gov). Two other mRNAs that exhibited refeeding-induced upregulation of ribosome density in the present study, i.e., *Pfkfb3* and *Ucp2*, are neither TOP nor TOP-like mRNAs. However, the *Ucp2* mRNA has a classical TOP motif beginning at nucleotide 9 in the sequence on the NCBI website, and based on information in the FANTOM5 Rat Cage database (https://fantom.gsc.riken.jp/zenbu/), the C residue at the beginning of the motif is a potential alternative transcriptional start site. Consequently, it is perhaps not surprising that refeeding a fasted rat would promote ribosome attachment to the *Ucp2* mRNAs. However, the 5’-untranslated region (UTR) of the *Pfkfb3* mRNA has neither a TOP nor a TOP-like motif based either on the sequence in the NCBI database or on the most common transcriptional start site in the FANTOM5 Rat Cage database, suggesting that its translation is regulated through a mechanism distinct from mRNAs with a TOP or TOP-like motif.

The mechanism through which mRNAs lacking a TOP or TOP-like motif are translationally regulated is unknown. However, in the present study it may relate to the lack of an effect of diet on refeeding-induced phosphorylation of 4E-BP1 (eukaryotic initiation factor 4E binding protein 1). In this regard, recent studies (36, 65) showed that in cells in culture deficient in both 4E-BP1 and 4E-BP2 (DKO), changes in mRNA translation induced by mTORC1 inhibitors, e.g., Torin1, are absent or significantly attenuated. Thus, the eIF4E binding proteins play a critical role in mediating many mTORC1-induced changes in mRNA translation. However, some mRNAs are still translationally up- or downregulated in DKO cells, suggesting that mTORC1 acts through additional mechanisms to regulation mRNA translation. Based on the results of the present study, it is tempting to speculate that changes in phosphorylation of rpS6 might be a 4E-BP-independent mechanism involved in refeeding-induced changes in mRNA translation. In addition, mTORC1-independent mechanisms cannot be ruled out.

In a recent study (29), we showed that refeeding-induced activation of mTORC1 was blunted in the liver of obesity-prone Sprague Dawley rats fed a HFD compared to a control diet for two weeks. However, in the present study, mTORC1 activation was similar in the livers of obesity-prone Sprague Dawley rats fed either a WD or a CD. The reason for the different response to refeeding in the two studies is unknown. However, the results from polysome profiling are consistent with mTORC1 activity being lower in the liver of WD compared to CD fed rats. Thus, the proportion of 46 TOP mRNAs in the heavy polysomal fraction was lower in the liver of rats fed a WD compared to a CD (**Supplemental Figure 4**). Moreover, refeeding-induced activation of mTORC1, as assessed by changes in phosphorylation of rpS6, was blunted in the liver of Sprague Dawley rats fed a WD compared to a CD in association with attenuated ribosome association of several TOP and TOP-like mRNAs.

More than 500 genes were translationally downregulated at least 1.5 log_2_(FC) in response following 1-h re-feeding. Interestingly, gene ontology analysis of these genes using Enrichr (https://maayanlab.cloud/Enrichr/) identified significant enrichment of genes associated with mitochondrial mRNA translation (e.g., *Mrpl27*, *Mrpl38*, *Mrps18A*, *Mrps21*, *Mrps34*, *Mrpl4*, *Mrpl13*, *Mrps6*, *Mrpl21*, *Ears2*). Moreover, functional analysis of these translationally downregulated genes, found significant enrichment of genes associated with mitogen-activated protein kinase binding (*Prmt1*, *Dusp8*, *Sirt1*, *Dusp6*), NAD-dependent histone deacetylase activity (*Sirt1*, *Sirt6*), and protein-arginine omega-N monomethyl transferase activity (*Prmt7* and *Prmt1*), pathways associated with perturbations in translation in previous studies [*Mapk* (66–70); *Hdac*/*Sirt* (71–76); *Prmt* (77–81)].

Overall, the results of the present study show that feeding a WD for as little as two weeks induced significant changes in both the transcriptome and the ribosome association of mRNAs in the liver. Many of the changes were in genes involved in lipid and carbohydrate metabolism, although genes involved in other processes were also identified. A number of mRNAs with TOP or TOP-like motifs in the 5’-UTR were identified in the polysome profiling portion of the study, but many others lack such structures and therefore are likely to be regulated through alternative mechanisms that need to be delineated in future studies.

## Data availability statement

The RNAseq datasets generated in this study can be found in the NCBI’s Gene Expression Omnibus (82), and are accessible through GEO Series accession number GSE214513 at https://www.ncbi.nlm.nih.gov/geo/query/acc.cgi?acc=GSE214513.

## Ethics statement

The animal study was reviewed and approved by Penn State College of Medicine Institutional Animal Care and Use Committee.

## Author contributions

JW, MD, LJ, and SK designed research; JW and HL conducted research; JW, YI, and SK analyzed data; and JW and SK wrote the paper. SK had primary responsibility for final content. All authors read and approved the final manuscript.

## Funding

This work was supported by NIH grant DK013499. This project was funded, in part, under a grant with the Pennsylvania Department of Health using Tobacco CURE Funds. The Department specifically disclaims responsibility for any analyses, interpretations, or conclusions.

## Acknowledgments

The authors would like to thank Drs. Ryan Hobbs for his assistance in OCT tissue sectioning and Anna Salzberg for assistance with RNAseq and Riboseq analyses.

## Conflict of interest

The authors declare that the research was conducted in the absence of any commercial or financial relationships that could be construed as a potential conflict of interest.

## Publisher’s note

All claims expressed in this article are solely those of the authors and do not necessarily represent those of their affiliated organizations, or those of the publisher, the editors and the reviewers. Any product that may be evaluated in this article, or claim that may be made by its manufacturer, is not guaranteed or endorsed by the publisher.
