# Supplementary figures and images for: An integrative approach to assessing effects of a short-term Western diet on gene expression in rat liver
Source: Front Endocrinol (Lausanne). 2022 Oct 26;13:1032293. doi: 10.3389/fendo.2022.1032293 (PMC9643360; doi:10.3389/fendo.2022.1032293)

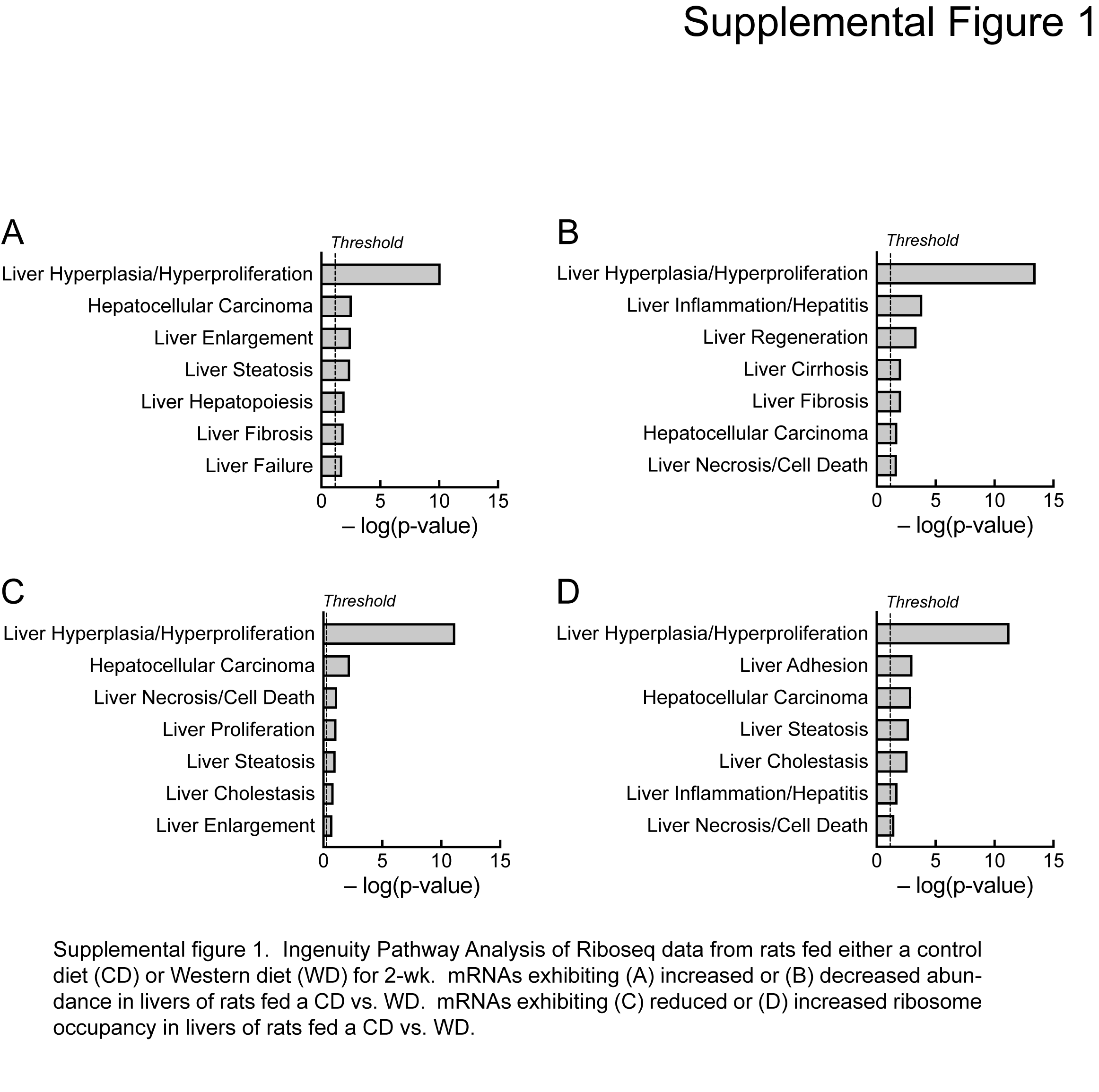

Supplement: Supplementary file 1 [file Image_1.tif]

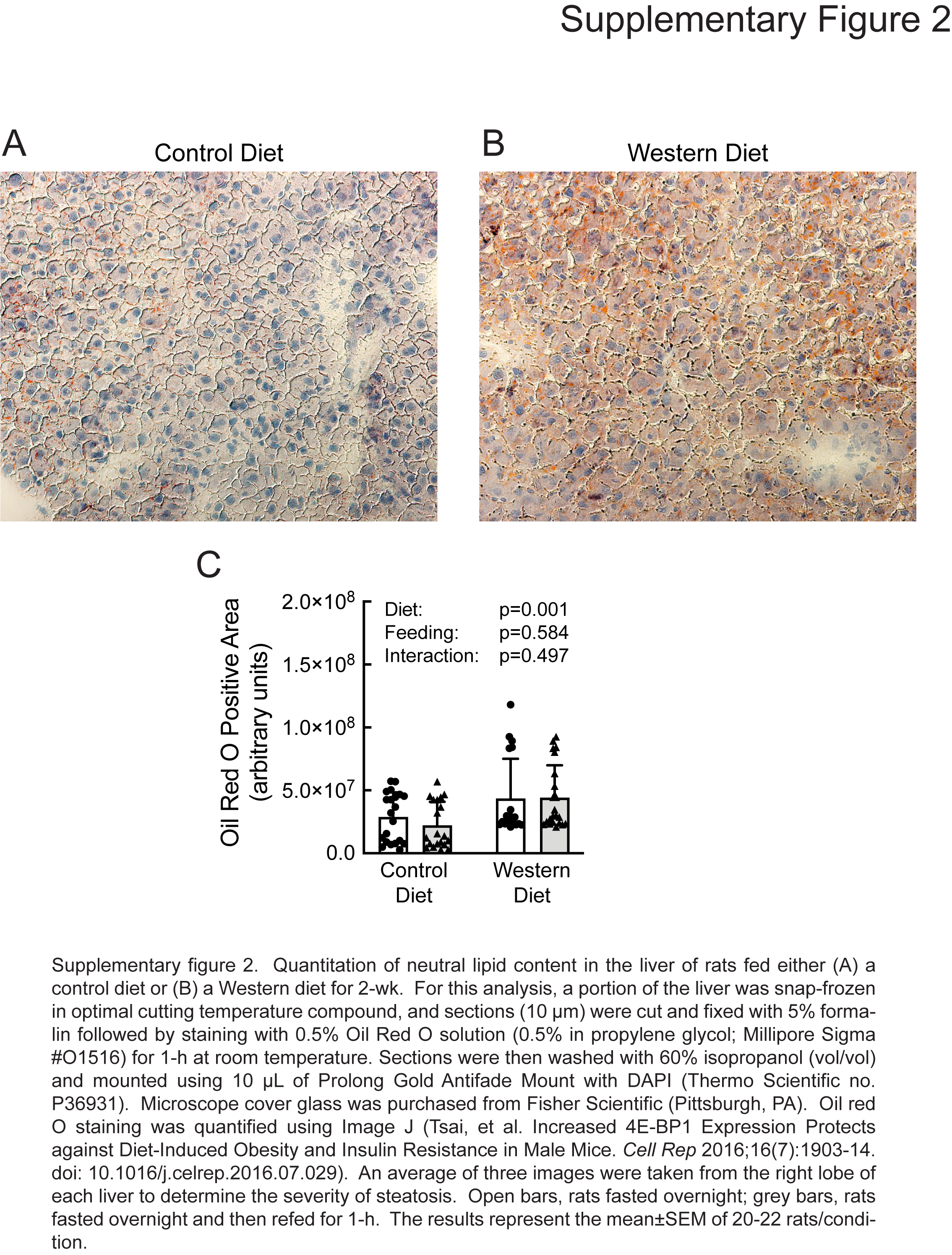

Supplement: Supplementary file 2 [file Image_2.tif]

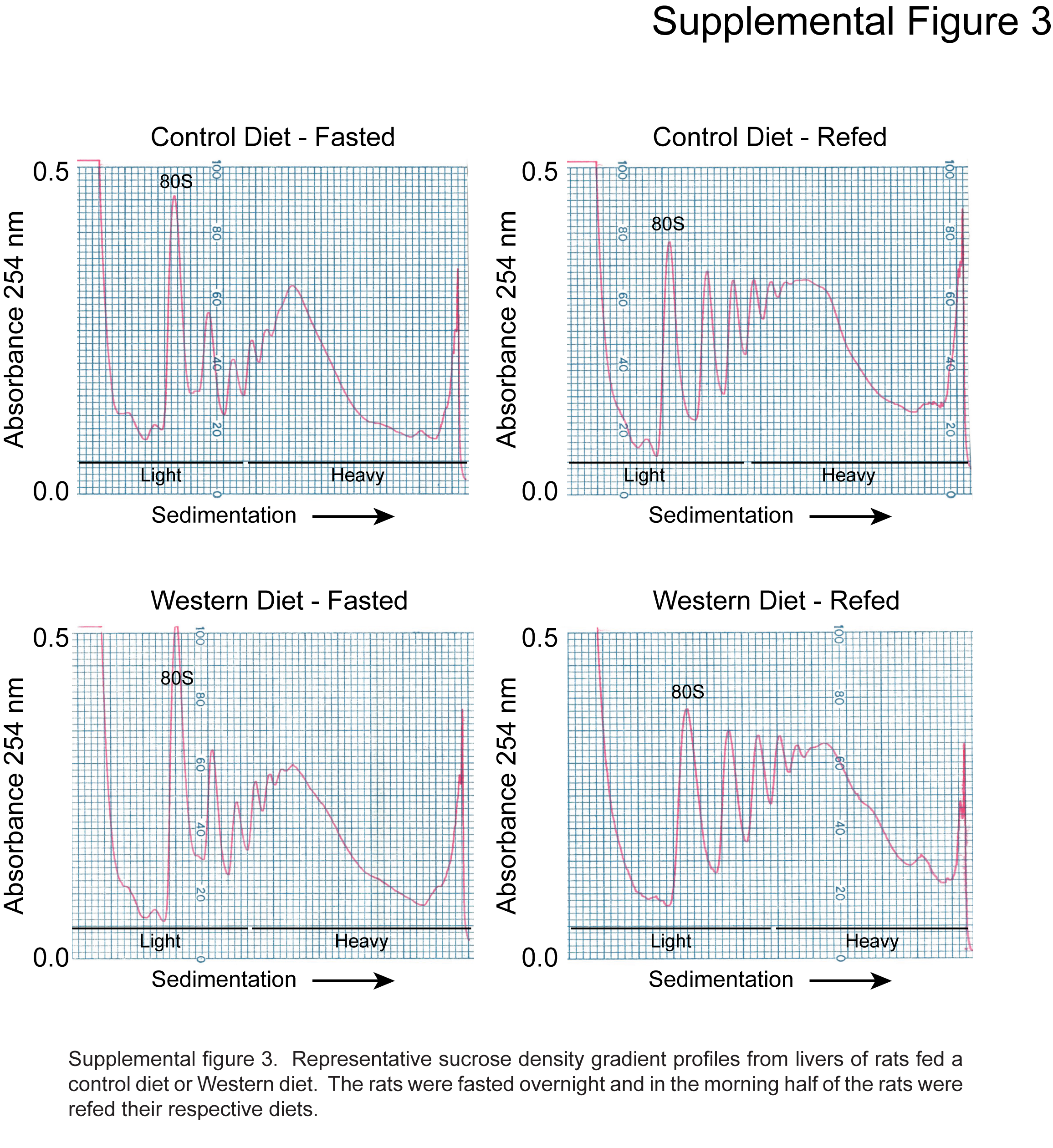

Supplement: Supplementary file 3 [file Image_3.tif]

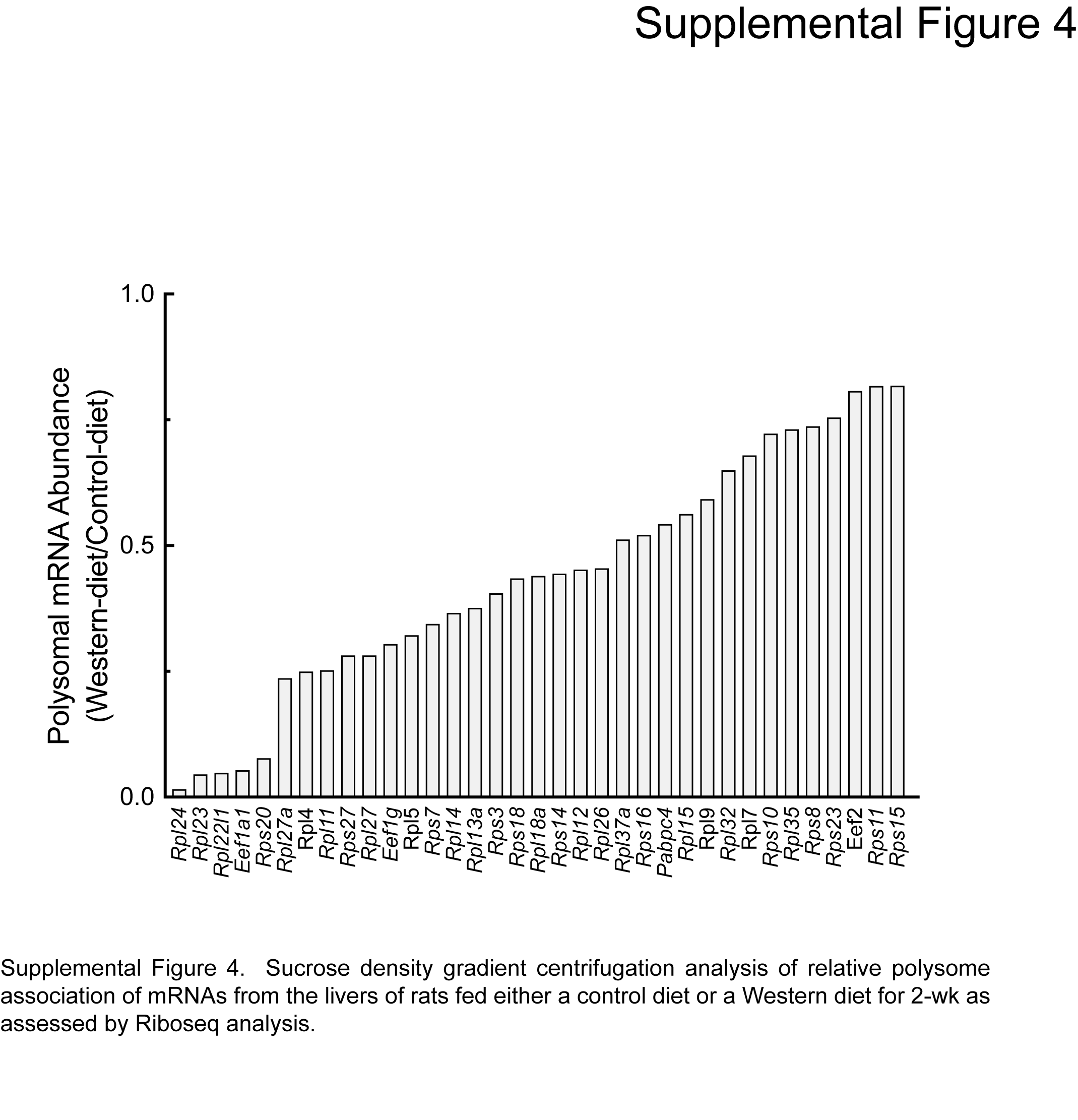

Supplement: Supplementary file 4 [file Image_4.tif]
