# Supplementary material for: An integrative approach to assessing effects of a short-term Western diet on gene expression in rat liver
Source: Front Endocrinol (Lausanne). 2022 Oct 26;13:1032293. doi: 10.3389/fendo.2022.1032293 (PMC9643360; doi:10.3389/fendo.2022.1032293)
Supplement: Supplementary file 5 [file Table_1.pdf]

Supplemental Table 1
Composition of Experimental Diets

| | Control Diet TD.08485 (g/Kg) | Western Diet TD.88137 (g/Kg) |
|---|---|---|
| Casein | 195 | 195 |
| DL-Methionine | 3 | 3 |
| Sucrose | 120 | 341.46 |
| Corn Starch | 432.99 | 150 |
| Maltodextrin | 100 | – |
| Anhydrous Milkfat | 37.2 | 210 |
| Cholesterol | – | 1.5 |
| Soybean Oil | 12.8 | – |
| Cellulose | 50 | 50 |
| Mineral Mix, AIN-76 | 35 | 35 |
| Calcium Carbonate | 4 | 4 |
| Vitamin Mix (Teklad 40060) | 10 | 10 |
| Ethoxyquin (antioxidant) | 0.01 | 0.04 |

| | Control Diet | | Western Diet | |
|---|---|---|---|---|
| | % by Weight | % by kcal | % by Weight | % by kcal |
| Protein | 17.3 | 19.1 | 17.3 | 15.2 |
| Carbohydrate | 61.3 | 67.9 | 48.5 | 42.7 |
| Fat | 5.2 | 13.0 | 21.2 | 42.0 |

| **Fatty Acid Profile (macro)** | **% of diet** |
|---|---|
| Saturated Fat | 12.8 |
| Monosaturated fat | 5.6 |
| Polyunsaturated fat | 1.0 |
| Unknown | 1.3 |
| **Fatty Acid Profile (micro)** | |
| Saturated fat | 61.8 |
| Monounsaturated fat | 27.3 |
| Polyunsaturated fat | 4.7 |
| 4:0 | 2.1 |
| 6:0 | 1.5 |
| 8:0 | 1.1 |
| 10:0 | 2.6 |
| 12:0 | 3.3 |

| | |
|---|---|
| 14:0 | 10.6 |
| 16:0 | 28.9 |
| 16:1 | 1.5 |
| 18:0 | 12.5 |
| 18:1 (oleic) | 20.9 |
| 18:1 isomers | 4.0 |
| 18:2 (linoleic) | 2.3 |
| 18.2 isomers | 1.3 |
| 18.3 (linolenic) | 0.7 |
