# Supplementary material for: An integrative approach to assessing effects of a short-term Western diet on gene expression in rat liver
Source: Front Endocrinol (Lausanne). 2022 Oct 26;13:1032293. doi: 10.3389/fendo.2022.1032293 (PMC9643360; doi:10.3389/fendo.2022.1032293)
Supplement: Supplementary file 6 [file Table_2.pdf]

Supplementary Table 2

Sequence of Primers used for Quantitative Real-Time PCR

Gene		Sequence (5'-3')
Lpl	Forward	TACCAAGCTGGTGGGAAATG
	Reverse	GCTGGATCCAAGCCAGTAAT
Lipe	Forward	CCCTCTACTCGTCACCCATA
	Reverse	GCAGGTCCTCTACCACTTTC
Pfkfb3	Forward	TGGAATTAGAGCGCCAAGAG
	Reverse	AGTGGGCATTTCAGGTATGG
Ucp2	Forward	GTCTGCACTCCTGTGTTCTC
	Reverse	CTCTAAAGGTGTCCCGTTCTTC
Eef1A	Forward	GATCGATCGTCGTTCTGGTAAG
	Reverse	AGTGGAGGGTAGTCAGAGAAG
Rps8	Forward	CGCATACATACAGTCCGAGTTC
	Reverse	CAGTTCTTCACCAGGGTCTTG
Map3k14	Forward	TGGAACTTCGGAGGAAAGTG
	Reverse	CTCTCTTGGCAGGGTATGTAAG

Supplementary Table 2

Antibodies Used for Western Blot Analysis

Antigen	Source, Item Number	Dilution
p70S6K	Bethyl Laboratories, #A300-510A	1:10,000
phospho-p70S6K (Thr389)	Cell Signaling Technology, #9205	1:1,000
Akt	Cell Signaling Technology, #4685	1:1,000
phospho-Akt (S473)	Cell Signaling Technology, #9271	1:1,000
rpS6	Cell Signaling Technology, #2317	1:1,000
phospho-rpS6 (S240/244)	Cell Signaling Technology, #2215S	1:1,000
4E-BP1	Cell Signaling Technology, #9644S	1:1,000
phospho-4E-BP1 (T37/46)	Cell Signaling Technology, #2855	1:1,000
Rabbit light chain HRP conjugate	Bethyl Laboratories, #A120-113P	1:10,000

Supplementary Table 3

mRNAs in the Ingenuity Pathway Analysis Category Liver Steatosis that were ≥1.5-fold Higher in Rats Fed a Western Diet Compared to a Control Diet*

Polysome Distribution (heavy/light)	
Gene Symbol	Expression Intensity/RPKM/FPKM/Counts
MAP3K14	25.472
UCP2	12.736
LIPE	11.321
PDE4C	8.491
PDE5A	6.368
CD14	4.776
FGF21	3.715
DNAJC7	3.569
Cyp4a14	3.393
HMOX1	3.216
MAP3K5	3.185
PDE7B	3.185
PANK2	3.184
PITPNA	2.952
ID1	2.830
MFSD2A	2.581
GCGR	2.502
UPP1	2.476
ABCG1	2.456
KRT8	2.444
AGPAT2	2.386
GNA11	2.377
MTHFR	2.286
PEMT	2.240
CLU	2.236
STEAP4	2.176
CRTC3	2.162
PKM	2.123
BAX	2.123
GBP2	2.123
TLR2	2.123
NCOA5	2.123
PPARGC1A	2.123
DGAT1	2.123
TERF2IP	2.004
LPL	1.930
RBPJ	1.916
LBP	1.914
ACADS	1.886
ELOVL5	1.885
CIDEB	1.862
THRA	1.859
CNR2	1.834
ELOVL2	1.833
IKBKB	1.819
PEX13	1.808
TM6SF2	1.789
BLVRA	1.769
STK25	1.722
MAPK8	1.701

mRNA Abundance	
Gene Symbol	Expression (fold-change)
CRTC3	8.364
DDIT3	3.535
PDGFC	3.139
SOAT1	3.137
IGFBP1	2.614
HMOX1	2.300
CD14	2.171
TERF2IP	2.091
IFNAR2	1.942
GATM	1.917
PEX2	1.882
RBL1	1.882
SIRT3	1.882
LBP	1.878
HSF1	1.830
PDE5A	1.792
BID	1.743
BLVRA	1.743
FDXR	1.743
NCOA5	1.743
LPL	1.699
FABP4	1.673
IKBKB	1.673
HINT2	1.596
NR4A1	1.591
FOXO1	1.568
MTHFR	1.568
AHR	1.532
PNRC2	1.524

*colored cells denote genes common to both lists

Supplementary Table 4

Sequence of the 5'-End of Select Rat mRNAs

Gene	Accession Number	5'-UTR	5'-UTR Length
Eef1a	NM_175838.2	5′-**tttttc**gcaa cgggtttgcc	66 nt
Rps8	NM_031706.1	5′-**ctctttcc**ag ccagcgccga	23 nt
Lipe	NM_012859.1	5′-tagccctaac ccctctccca	193 nt
Lpl	NM_012598.2	5′-**ctcctcc**aag aaattctgcc	189 nt
Pfkfb3	NM_057135.1	5′-gaggctgacg cgccgcgcta	405 nt
Ucp2	NM_019354.3	5′-actgtcagcc cctccctcgg	373 nt

Characters in bold denote terminal oligopyrimidine (TOP) motifs. Underlined characters denote uninterrupted stretches of four or more pyrimidine residues within 20 nucleotides of the 5'-end of the mRNA. UTR, untranslated region.
