# Supplementary material for: An integrative approach to assessing effects of a short-term Western diet on gene expression in rat liver
Source: Front Endocrinol (Lausanne). 2022 Oct 26;13:1032293. doi: 10.3389/fendo.2022.1032293 (PMC9643360; doi:10.3389/fendo.2022.1032293)
Supplement: Supplementary file 7 [file Table_3.pdf]

Supplementary Table 3

Antibodies Used for Western Blot Analysis

Antigen	Source, Item Number	Dilution
p70S6K	Bethyl Laboratories, #A300-510A	1:10,000
phospho-p70S6K (Thr389)	Cell Signaling Technology, #9205	1:1,000
Akt	Cell Signaling Technology, #4685	1:1,000
phospho-Akt (S473)	Cell Signaling Technology, #9271	1:1,000
rpS6	Cell Signaling Technology, #2317	1:1,000
phospho-rpS6 (S240/244)	Cell Signaling Technology, #2215S	1:1,000
4E-BP1	Cell Signaling Technology, #9644S	1:1,000
phospho-4E-BP1 (T37/46)	Cell Signaling Technology, #2855	1:1,000
Rabbit light chain HRP conjugate	Bethyl Laboratories, #A120-113P	1:10,000
