# Supplementary material for: An integrative approach to assessing effects of a short-term Western diet on gene expression in rat liver
Source: Front Endocrinol (Lausanne). 2022 Oct 26;13:1032293. doi: 10.3389/fendo.2022.1032293 (PMC9643360; doi:10.3389/fendo.2022.1032293)
Supplement: Supplementary file 8 [file Table_4.pdf]

Supplementary Table 4

mRNAs in the Ingenuity Pathway Analysis Category Liver Steatosis that were ≥1.5-fold Higher in Rats Fed a Western Diet Compared to a Control Diet*

Polysome Distribution (heavy/light)	
Gene Symbol	Expression Intensity/RPKM/FPKM/Counts
MAP3K14	25.472
UCP2	12.736
LIPE	11.321
PDE4C	8.491
PDE5A	6.368
CD14	4.776
FGF21	3.715
DNAJC7	3.569
Cyp4a14	3.393
HMOX1	3.216
MAP3K5	3.185
PDE7B	3.185
PANK2	3.184
PITPNA	2.952
ID1	2.830
MFSD2A	2.581
GCGR	2.502
UPP1	2.476
ABCG1	2.456
KRT8	2.444
AGPAT2	2.386
GNA11	2.377
MTHFR	2.286
PEMT	2.240
CLU	2.236
STEAP4	2.176
CRTC3	2.162
PKM	2.123
BAX	2.123
GBP2	2.123
TLR2	2.123
NCOA5	2.123
PPARGC1A	2.123
DGAT1	2.123
TERF2IP	2.004
LPL	1.930
RBPJ	1.916
LBP	1.914
ACADS	1.886
ELOVL5	1.885
CIDEB	1.862
THRA	1.859
CNR2	1.834
ELOVL2	1.833
IKBKB	1.819
PEX13	1.808
TM6SF2	1.789
BLVRA	1.769
STK25	1.722
MAPK8	1.701

mRNA Abundance	
Gene Symbol	Expression (fold-change)
CRTC3	8.364
DDIT3	3.535
PDGFC	3.139
SOAT1	3.137
IGFBP1	2.614
HMOX1	2.300
CD14	2.171
TERF2IP	2.091
IFNAR2	1.942
GATM	1.917
PEX2	1.882
RBL1	1.882
SIRT3	1.882
LBP	1.878
HSF1	1.830
PDE5A	1.792
BID	1.743
BLVRA	1.743
FDXR	1.743
NCOA5	1.743
LPL	1.699
FABP4	1.673
IKBKB	1.673
HINT2	1.596
NR4A1	1.591
FOXO1	1.568
MTHFR	1.568
AHR	1.532
PNRC2	1.524

*colored cells denote genes common to both lists
