# Supplementary material for: An integrative approach to assessing effects of a short-term Western diet on gene expression in rat liver
Source: Front Endocrinol (Lausanne). 2022 Oct 26;13:1032293. doi: 10.3389/fendo.2022.1032293 (PMC9643360; doi:10.3389/fendo.2022.1032293)
Supplement: Supplementary file 9 [file Table_5.pdf]

Supplementary Table 5

Sequence of the 5'-End of Select Rat mRNAs

Gene	Accession Number	5'-UTR	5'-UTR Length
Eef1a	NM_175838.2	5'-**tttttc**gcaa cgggtttgcc	66 nt
Rps8	NM_031706.1	5'-**ctctttcc**ag ccagcgccga	23 nt
Lipe	NM_012859.1	5'-tagccctaac ccctctccca	193 nt
Lpl	NM_012598.2	5'-**ctcctcc**aag aaattctgcc	189 nt
Pfkfb3	NM_057135.1	5'-gaggctgacg cgccgcgcta	405 nt
Ucp2	NM_019354.3	5'-actgtcagcc cctccctcgg	373 nt

Characters in bold denote terminal oligopyrimidine (TOP) motifs. Underlined characters denote uninterrupted stretches of four or more pyrimidine residues within 20 nucleotides of the 5'-end of the mRNA. UTR, untranslated region.
